# Supplementary material for: Causal Relationship between Aging and Anorexia Nervosa: A White-Matter-Microstructure-Mediated Mendelian Randomization Analysis
Source: Biomedicines. 2024 Aug 16;12(8):1874. doi: 10.3390/biomedicines12081874 (PMC11351342; doi:10.3390/biomedicines12081874)
Supplement: Supplementary file 1 [file biomedicines-12-01874-s001.zip › Supplementary Figure/Figure S4/Singe SNP plot of MR analysis for TL to ubm-b-2072.pdf]

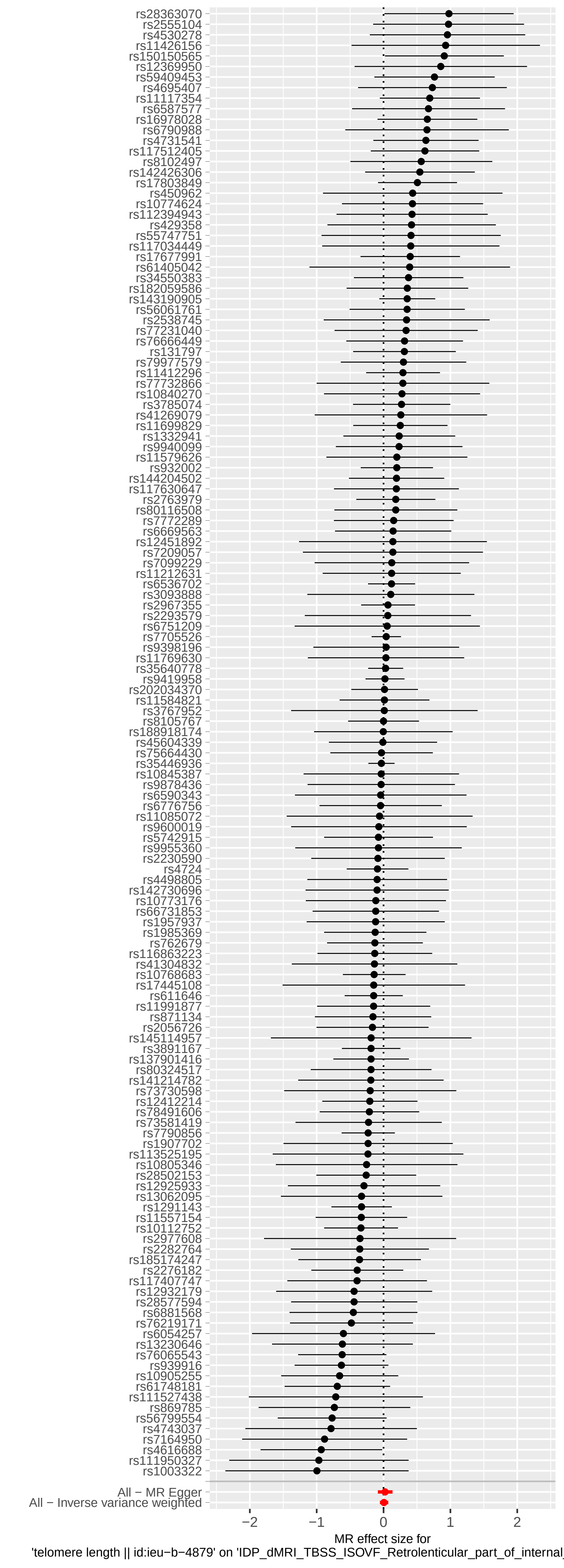
rs28363070
rs2555104
rs4530278
rs11426156
rs150150565
rs12369950
rs59409453
rs4695407
rs11117354
rs6587577
rs16978028
rs6790988
rs4731541
rs117512405
rs8102497
rs142426306
rs17803849
rs450962
rs10774624
rs112394943
rs429358
rs55747751
rs117034449
rs17677991
rs61405042
rs34550383
rs182059586
rs143190905
rs56061761
rs2538745
rs77231040
rs76666449
rs131797
rs79977579
rs11412296
rs77732866
rs10840270
rs3785074
rs41269079
rs11699829
rs1332941
rs9940099
rs11579626
rs932002
rs144204502
rs117630647
rs2763979
rs80116508
rs7772289
rs6669563
rs12451892
rs7209057
rs7099229
rs11212631
rs6536702
rs3093888
rs2967355
rs2293579
rs6751209
rs7705526
rs9398196
rs11769630
rs35640778
rs9419958
rs202034370
rs11584821
rs3767952
rs8105767
rs188918174
rs45604339
rs75664430
rs35446936
rs10845387
rs9878436
rs6590343
rs6776756
rs11085072
rs9600019
rs5742915
rs9955360
rs2230590
rs4724
rs4498805
rs142730696
rs10773176
rs66731853
rs1957937
rs1985369
rs762679
rs116863223
rs41304832
rs10768683
rs17445108
rs611646
rs11991877
rs871134
rs2056726
rs145114957
rs3891167
rs137901416
rs80324517
rs141214782
rs73730598
rs12412214
rs78491606
rs73581419
rs7790856
rs1907702
rs113525195
rs10805346
rs28502153
rs12925933
rs13062095
rs1291143
rs11557154
rs10112752
rs2977608
rs2282764
rs185174247
rs2276182
rs117407747
rs12932179
rs28577594
rs6881568
rs76219171
rs6054257
rs13230646
rs76065543
rs939916
rs10905255
rs61748181
rs111527438
rs869785
rs56799554
rs4743037
rs7164950
rs4616688
rs111950327
rs1003322
All − MR Egger
All − Inverse variance weighted
−2
−1
0
1
2
MR effect size for
'telomere length || id:ieu−b−4879' on 'IDP_dMRI_TBSS_ISOVF_Retrolenticular_part_of_internal_
